# Supplementary material for: Are carbon emissions trading and green financial instruments synergistic? -Comprehensive quantitative research based on content analysis
Source: PLoS One. 2024 Mar 7;19(3):e0298601. doi: 10.1371/journal.pone.0298601 (PMC10919601; doi:10.1371/journal.pone.0298601)
Supplement: S1 Data — (ZIP) [file pone.0298601.s001.zip › Data/地级市Z_F工作报告.zip!/║╙─╧/╓ú╓▌╩╨/└┤╫╘╣1⁄2╓┌║┼í╛┬φ┐╦╩2╛▌═°í┐_2019.docx]

各位代表：

现在，我代表市人民政府向大会报告工作，请予审议，并请各位政协委员和列席人员提出意见。

一、2018年工作回顾

刚刚过去的一年，是郑州发展史上具有里程碑意义的一年。一年来，我们在省委、省政府和市委的坚强领导下，高举习近平新时代中国特色社会主义思想伟大旗帜，按照党的十九大作出的战略部署，全面贯彻落实习近平总书记视察河南、郑州时的重要指示精神，树牢“四个意识”，坚定“四个自信”，做到“两个维护”，统筹推进“四个着力”，持续打好“四张牌”，有效应对各种困难风险挑战，砥砺奋进，拼搏赶超，较好完成了市十五届人大一次会议确定的主要目标任务。以地区生产总值破万亿、常住人口破千万、人均生产总值破10万“三大突破”为标志，出彩郑州站在了高质量发展的新起点。

（一）抢抓国家中心城市建设机遇，引领辐射作用日益凸显

国家中心城市建设迈出坚实步伐。省委、省政府支持郑州建设国家中心城市的意见即将出台，全面落实《郑州建设国家中心城市行动纲要（2017-2035年）》，总投资4.5万亿元的国家中心城市建设重大项目库发布，1000亿元产业发展基金设立运营，“一中枢一门户三中心”加快构建；上榜“国家物流枢纽承载城市”，陆港型、空港型、生产服务型、商贸服务型物流枢纽地位巩固提升；首次进入“世界城市100强”“亚洲城市50强”；中国社科院研究机构发布的“国家中心城市指数”中，我市荣登潜在国家重要中心七项榜单；“中国大陆最佳商业城市”和《中国金融中心指数报告》，我市均居第12位。区域协调发展新格局加快构建。晋身国家区域协调发展新机制12城市，《郑州大都市区空间规划（2018-2035年）》省委、省政府审议通过，发展目标、战略路径、主要任务进一步明晰；郑开双创走廊、开港产业带、许港产业带等6个专项规划加快报审，郑焦6个专项合作协议签订实施，郑新融合发展战略规划省政府审议通过，“1+4”郑州大都市区的规划、交通、产业等领域加速融合。经济贡献度进一步提升。以占全省4.45%的地域面积、10.55%的人口，创造了占全省21.1%的GDP、30.6%的一般公共预算收入、20.7%的社会消费品零售总额、32.9%的重点项目投资、74.5%的进出口总值，航空港实验区进出口总额突破500亿美元，占全省的62.8%；引进境内外资金356.7亿美元，其中实际吸收外资42.1亿美元，占全省的23.6%。

（二）始终坚持“稳”字当头，经济质量效益稳步提升

坚持从供给侧精准发力，保持了经济运行总体平稳、稳中有进的良好态势。主要指标稳中提质。初步核算，全市地区生产总值完成10143.3亿元，居全国298个地级以上城市第16位，比上年前移1位；增长8.1%，居全国16个万亿以上城市第2位。规模以上工业增加值增长6.8%；工业投资增长11.8%，扭转了近年来持续下滑态势，高载能产业占比首次下降到40%以下。固定资产投资增长10.9%；社会消费品零售总额增长9.7%；地方财政一般公共预算收入达到1152.1亿元，增长9%，税收占比达到74.6%，高出全省4个百分点。新增市场主体25.2万户，总量达到107.5万户，成为第8个超百万的省会城市。民营经济占GDP的比重达到58.9%，从业人员占城镇就业人员的比重达到87.3%。单位生产总值能耗降低率达到7%，万元工业增加值能耗下降14.8%。现代产业体系加快构建。高技术产业增加值增长12.4%，战略性新兴产业增加值占规模以上工业比重达到20%，新能源客车产销量突破2.5万辆、占全国的27%。七大主导产业增加值增长7.6%，对全市工业增长的贡献率达到93.9%，初步形成电子信息、汽车及装备制造两个5000亿级产业集群，国家级技术创新和制造业单项冠军示范企业达到9家。服务业增加值5545.5亿元、增长8.3%。旅游接待总人数1.15亿人次、总收入突破1300亿元，分别增长13.6%、14.3%，卢森堡旅游签证（郑州）便捷服务平台投运，郑东新区中央商务区成为“中国最具活力中央商务区”之一。金融业增加值1145.8亿元、占GDP的11.3%，郑州农商行获批筹建，郑州银行成为全国首家A+H股上市的城商行。建筑业增加值增长16.5%；物流业增加值780亿元、增长9%，国际物流园区晋升为国家级示范物流园区。新增环城都市生态农业15万亩，农业产业化龙头企业10家。 “智能制造”提速增效。 “三大改造”深入推进，工业技改投资增长35.4%，制造业投资占工业投资比重达到79.5%，战略性新兴产业投资增长26.4%，其中高端装备制造业、新一代信息技术产业投资分别增长380.5%、734.7%。新增超百亿企业2家、超200亿企业1家，“百千企业上云”近4700家。成功举办首届世界传感器大会、中国（郑州）承接产业转移系列对接活动等，引进上汽二期等一批重大制造业项目，中铁智能装备产业园等206个项目开工建设，合晶一期等211个项目竣工。成功创建国家服务型制造示范城市、中国消费品工业“三品”战略示范城市，中牟汽车产业集聚区成为国家新型工业化产业示范基地。

（三）强化科技创新引领，高质量发展新动能加快培育

聚焦“四个一批”，促进“四个融合”，万人发明专利拥有量13件、增长20%，科技进步贡献率达到63%。自创区建设步伐加快。大数据产业园等重大项目入驻中原科创谷，高新区管理体制和人事薪酬制度改革经验将在全省国家级开发区推广；全省首个新型产业用地试点成功落地。创新创业载体达211家，孵化载体面积突破850万平方米，在孵企业（团队）近万家，孵化水平居全国13位。技术合同交易额82.3亿元，增长136.8%，全省占比54.9%。 “四个一批”成效明显。高新技术企业和科技型企业分别达到1329家、4283家，增长55%、32.7%；大力实施“智汇郑州”人才工程，吸引21万余名青年人才来郑就业创业，办理人才落户近5万人；成功承办首届中国·河南招才引智创新发展大会，签约项目111个，引进高层次人才293人。科技创新实现重大突破。积极推动协作创新，实施通信技术、超级电容等十大科技专项，8项成果获2018年度国家科技奖励，创历史新高。旭飞光电参与研发的光电显示项目获国家科技进步一等奖，结束了我国玻璃基板完全依赖进口的历史；中铁装备公司研发的异形全断面掘进机获二等奖，填补了国内外技术空白；盾构及掘进技术实验室被科技部评为全省唯一优秀国家重点实验室。新增省级以上研发中心221家、制造业创新中心3个。

（四）坚定不移推进改革开放，发展动力活力持续增强

重点改革加快推进。政府机构改革全面启动，因地制宜设置40个政府工作部门，新老机构顺利交替。“放管服”改革持续深化。在全省率先启动“最多跑一次”改革，市级政务服务办事大厅建成投用，878个审批服务事项分类“一窗受理”，全面推行一次告知、“肩并肩”辅导等机制，为群众提供了“门好进、脸好看、事好办”的政务服务。投融资体制改革持续深化。财政资金基金化改革有序推进，市场准入负面清单试点加快实施，公共资源交易在线监管全覆盖。国企改革攻坚行动强力实施。13家市管工业企业改制大头落地，34家“僵尸企业”实质出清，市管三级企业混改比例达47%。农村土地承包经营权确权颁证全面完成，农村集体产权制度改革扎实推进；社会信用体系建设荣获国家“守信激励创新奖”，城市管理综合执法、医疗卫生等领域改革加快推进。 “五区联动”综合发力。航空港实验区电子信息先进制造业集群向高端拓展，电子信息产业产值突破3000亿元，“双创”示范基地受到国务院表彰。自贸区郑州片区256项改革创新试点任务已超前完成五年计划的86.7%，新增注册企业4万家、占河南自贸区的80.6%。大数据综试区加快建设，中科院计算所郑州分所挂牌成立，累计引进大数据及相关企业200余家。口岸建设成效突出，汽车平行进口试点、中欧班列运邮试点城市获批，进境粮食指定口岸试运营，内陆地区口岸数量最多、种类最全的城市地位继续保持。 “四路协同”效应显现。 “空中丝绸之路”越飞越广，郑州机场获批第五航权，已开通航线236条，横跨欧美亚三大经济区国际枢纽航线网络初步形成，成为全国第二个实现航空、铁路、轨道交通、高速公路一体化换乘机场。“陆上丝绸之路”越跑越快，中欧班列（郑州）每周“去九回八”高频次运行，新开通中亚、东盟线路，全年开行752班，主要指标继续保持全国前列。“网上丝绸之路”越来越便捷，EWTO核心功能集聚区启动建设，新签约项目45个，第二届全球跨境电子商务大会成功举办，跨境电商交易额增长25.1%。“海上丝绸之路”越来越顺畅，郑州至连云港、青岛、天津等港口海铁联运班列累计开行206班。

（五）集中精力打好三大攻坚战，补短板强弱项取得积极进展

始终把“三大攻坚战”作为政治任务盯紧抓牢。防范化解重大风险攻坚战扎实推进。全面开展金融机构不良资产压降、非法违规金融活动打击、互联网金融风险和政府隐性债务“四清四实”专项整治行动，依法依规处置非法集资事件，稳妥处置重点领域个案重大风险，有序化解上市公司流动性危机，政府债务管理机制进一步规范。精准脱贫攻坚加快推进。脱贫退出1434户3800人，占全年目标的122%，剩余贫困人口1785人，实现除政策兜底外全部脱贫，脱贫攻坚由取得决定性进展向夺取全面胜利转变。结对帮扶贫困县工作深入开展，22项重点工作和197个帮扶项目启动。全面打好污染防治攻坚战。认真做好中央环保督察“回头看”及省委、省政府环保督察交办问题整改工作，制定实施大气、水、土壤污染防治攻坚三年行动计划。围绕“四大结构”调整，深入开展工业企业提标改造、柴油货车污染治理、燃煤用量削减、城乡结合部环境综合整治、扬尘治理等专项行动，强力推进秋冬季大气污染攻坚，全市空气质量明显改善。2018年7、8、9连续三个月空气质量首次达到国家二级标准，综合指数、6项指标、优良天数实现“七降一增”，为2013年执行国家空气质量新标准以来最好水平；PM10、PM2.5年均浓度同比分别下降10.2%、4.5%，超额完成国家和省定目标。坚持“治、护、建、管”并重，全面推行河（湖）长制，大力实施 “四水同治”工程，6个国省控断面实现水质类别提升，通过国家节水型城市建设复查和全国水生态文明城市建设试点验收、集中式饮用水水源地专项排查验收，城市黑臭水体整治获得国家督查组肯定。完善污染地块土壤环境管理联动机制，加快推进土壤治理与修复省级试点项目，完成重点行业企业土壤污染状况调查，土壤污染防治攻坚战有序推进。

（六）健全完善城市功能体系，城乡融合发展持续深化

国家综合交通枢纽地位进一步强化。我市被确定为全国12个最高等级国际性综合交通枢纽之一。《郑州国际航空货运枢纽战略规划（2018-2035年）》《郑州铁路枢纽总图规划（2016-2030年）》成功获批，《畅通郑州白皮书（2019-2021年）》编制完成。郑济、郑万、郑阜高铁及郑州南站、机场至南站城际铁路加快建设。高铁客流量发送3303.8万人次、增长14.9%；航空货邮吞吐量51.3万吨、客运量2733.5万人次，分别居全国大型机场第7、第12位。机西高速二期、商登高速（郑州段）建成通车，新建改建国省干线和农村公路320公里，环城高速出入口达到24个。地铁5号线空载试运行，轨道交通运营及在建里程突破300公里。中心城区“两横两纵+环线”快速路网体系基本形成，成功创建“国家公交都市建设示范城市”。 城市基础设施加快建设。百城建设提质工程深入实施，统筹做好“四篇文章”，抓好城市“四治”，实施项目1461个，完成投资2182亿元。“四大文化片区”有序推进，中央文化区（CCD）“四个中心”基本完工。新建公共停车泊位5.2万个，新建改造公厕1692座，综合管廊、清洁取暖等国家试点有序推进，新增供热面积1600万平方米，新增生活污水日处理能力20万吨，日处理4000吨的垃圾发电厂和600吨的餐厨垃圾处理厂即将投用。市县两级城市建成区面积830平方公里，全市城镇化率达73.4%。城市精细化管理三年行动计划全面启动。环卫保洁标准大幅提升，城市管理范围从三环内拓展至近郊48个乡镇，面积达到1440平方公里。四环内全面推行三级 “路长制”，管理责任落实到了最后一米、最后一人；实施道路大中修155条、支路背街改造提升71条；清理清除楼顶广告6521个、路边路牌广告、道旗广告4056杆。入选全国35个建筑垃圾治理试点城市，城市生活垃圾分类工作进入国家46个试点城市前10位。生态建设成效明显。强力推进国土绿化提速行动，铁路沿线、干线公路、高速立交及出入市口等区域违建全部拆除，提质连通生态廊道528公里，新造林11.5万亩，完成森林抚育7.8万亩；市区新增绿地1356万平方米，建成高铁公园等公园、游园、微公园411个。第十一届园博会圆满闭幕，园博园常态化开放。贾鲁河综合治理蓝线工程基本完工，绿线工程全面启动。牛口峪引黄等三大水源工程主体建成，河道采砂整治成效进一步巩固，全年向城区河道调水3.8亿立方米。乡村振兴战略深入推进。27个美丽乡村和364个规划保留村生活污水集中处理设施加速推进，1037个基层综合性文化服务中心、148个乡镇公厕改造竣工投用。“大棚房”专项整治成效明显。非洲猪瘟等动物疫病防控扎实有效。

（七）聚焦民生福祉下功夫，人民群众获得感幸福感不断增强

坚持在发展中补齐民生短板，省市民生实事基本完成。全年财政民生支出1402.9亿元、增长17%，占全市财政一般公共预算支出的79.6%。聚焦“一老一小”提供优质服务。高度关注养老问题，全市养老床位达到4万张，城乡居民月人均养老待遇达到239元。全域通过国家义务教育发展基本均衡工作评估。幼儿园建成投用37所，新建、改扩建中小学38所，投用20所，大班额、上学难问题逐步缓解。聚焦就业消费提高群众收入。新增城镇就业12.4万人、农村劳动力转移就业6.12万人，“零就业家庭”动态为零。居民消费价格指数上涨2.4%，“衣食住行”等八大类商品价格均保持温和上涨。居民人均可支配收入完成33105元，增长8.3%，城乡居民收入比缩小到1.8。专业市场转型升级和有序疏解步伐加快。聚焦就医住房缓解群众难题。公立医院综合改革受到国务院表彰，药品、高值耗材联合采购实现全覆盖，药占比29.6%，各医疗机构药品“两票制”执行比例98%以上，“家门口医疗服务”更加便捷；基本建成保障性住房9.4万套，首批2.5万套青年人才公寓开工建设，安置房网签12万套，新增回迁群众30万人、回迁率84%。房地产市场保持平稳。聚焦文化惠民满足群众需求。公共文化服务体系进一步完善，服务效能不断提升，文化艺术和产业提质发展。成功创建国家首批文化消费试点城市，成为全国最爱阅读城市之一。成功举办戊戌年黄帝故里拜祖大典、第十二届中国郑州国际少林武术节、2018郑州航展、2018郑州国际马拉松赛等活动赛事，第十一届全国少数民族传统体育运动会筹备工作有序推进。

在做好各项工作的同时，我们聚焦聚力作风建设，不折不扣落实党中央、国务院决策部署和省委、省政府及市委要求，大力营造真抓实干、拼在一线的强大气场。我们夯实全面从严治党主体责任，不断加强民主法治建设、党风廉政建设和政府系统党的建设，深入推进依法行政，强化审计监督，持续纠正“四风”，认真做好中央、省委巡视及国务院大督查反馈问题整改工作，自觉执行市人大决议决定，主动接受各方监督，385件人大代表建议（议案）、683件政协提案全部办结。推进军民融合深度发展，“双拥”工作持续深化。深入开展“扫黑除恶”专项斗争，依法打击各类违法犯罪和暴力恐怖活动。民族宗教工作创新推进。安全生产、食品药品安全、信访稳定、社会治理等工作进一步加强。妇女儿童、残疾人、红十字、慈善等事业健康发展。外事、侨务、对台、统计、史志、气象、地震、社科研究、援疆等工作取得新成绩。

各位代表，过去一年取得的成绩，是以习近平同志为核心的党中央掌舵领航的结果，是省委、省政府和市委坚强领导的结果，是中央、省驻郑单位共同参与、市人大市政协支持监督和全市上下奋力拼搏的结果。在此，我代表市人民政府，向全市人民，向给予政府工作大力支持的人大代表、政协委员，向驻郑人民解放军、武警官兵，向各民主党派、工商联、各人民团体及各界人士，向参与郑州建设的全体劳动者，向关心支持郑州发展的港澳台同胞、海外侨胞、国际友人，致以崇高的敬意和诚挚的感谢！

同时，我们也清醒认识到，我市经济社会发展还存在许多不平衡不充分的矛盾和问题。主要是：产业发展质量不高，传统产业仍占较大比重，新兴产业增速快但体量小，金融和实体经济的良性循环尚未形成，投资特别是民间投资不足。科技创新能力不强，企业研发投入比例不高，科技成果转化有待加强，高端创新人才、创新团队仍然偏少。生态环境保护任务依然繁重，产业结构、能源结构、交通运输结构不优，大气、水体、土壤环境治理压力较大，绿色低碳生产生活方式还需加快推进。民生保障还存在不少短板，就业、教育、医疗、住房、养老、家政等方面与群众期待还有差距，影响社会稳定的突出矛盾需要加快解决。政府治理体系和治理能力与国际化现代化生态化大都市要求还有差距，营商环境有待优化，城市管理精细化水平还需提高，一些领域不正之风和腐败问题时有发生等。对此，我们将不畏难、不避险，采取有力措施，切实改进解决。

二、2019年政府工作总体要求和主要预期目标

2019年是新中国成立70周年，也是推动国家中心城市高质量建设、决胜全面建成小康社会、实现第一个百年奋斗目标的关键之年。综合判断，今年有挑战更有机遇，有压力更有动力。

——站在国家中心城市高质量建设新的历史起点，我们要有强烈的使命意识。2014年5月习近平总书记视察河南和郑州时，提出郑州要“建成连通境内外、辐射东中西的物流通道枢纽，为丝绸之路经济带多作贡献”，向“买全球卖全球”目标迈进、打造中部地区对外开放高地。2016年12月国家明确提出支持郑州建设国家中心城市，2018年11月晋身国家区域协调发展新机制12个城市。省委、省政府全力支持郑州国家中心城市建设，强调在引领中原城市群发展中，“让郑州这个龙头高高扬起来”。我们要始终牢记习近平总书记的殷殷嘱托和省委、省政府的希望重托，把“引领区域发展、参与国际竞争、代表国家形象、服务国家战略”作为郑州建设国家中心城市的方位、目标和使命，推动经济高质量发展、城市高品位建设，努力在全国全省大局中贡献“郑州力量”。

——站在国家中心城市高质量建设新的历史起点，我们要有强烈的机遇意识。当前，建设国家中心城市是我市最大发展机遇，航空港实验区、自贸区郑州片区、郑洛新国家自主创新示范区等一大批国家战略和载体平台加快实施，政策叠加优势加速释放。中央继续实施积极财政政策和稳健货币政策，实施更大规模减税降费，加大基础设施领域补短板力度，有利于我们抢抓机遇加快发展。新一轮科技革命和产业变革蓬勃兴起，“四新”经济不断涌现，只要主动作为，就能抢占先机。作为1亿人口大省的省会，“1+4”郑州大都市区空间规划深入实施，“枢纽+区位”的优势地位更加巩固，将释放巨大的发展潜力和市场空间。特别是近年来在市委坚强领导下，全市上下形成了“心齐、劲足、气顺”谋发展的浓厚氛围。我们要抢抓机遇，深耕细作，勇作先行者，当好排头兵。

——站在国家中心城市高质量建设新的历史起点，我们要有强烈的忧患意识。世界经济下行风险逐步加大，中美贸易摩擦带来的经济不确定性增加，市场需求放缓，增长动能减弱；我市经济运行稳中有变、变中有忧，稳定经济增长的任务更加繁重；区域竞争更加激烈，追兵逼近、标兵奋进，不进则退、慢进亦退。我们要切实防范化解经济发展、政治安全、社会稳定、自然灾害、公共事件、安全生产等领域重大风险，牢固树立底线思维，打好主动仗、下好先手棋、增创新优势，以破解难题的成效带动全局工作提升，始终保持郑州在全国省会城市中晋位升级态势。

——站在国家中心城市高质量建设新的历史起点，我们要有强烈的担当意识。时刻牢记“新时代是奋斗者时代”的召唤，培养斗争精神和争先意识，面对使命敢于踏平坎坷，面对矛盾敢于迎难而上，面对危机敢于挺身而出，面对失误敢于承担责任，面对歪风邪气敢于坚决斗争，始终把准大势、顺应趋势，走对路子、踩准鼓点。只要有利于发展、有利于人民幸福，就毫不犹豫地大胆闯、勇于改、超前干，全力跑好郑州发展这一棒，用扎实业绩开创高质量发展新境界。

——站在国家中心城市高质量建设新的历史起点，我们要保持战略定力。紧盯国家中心城市高质量建设总目标，牢牢把握“稳中求进”总基调，突出“奋发有为”总要求，始终坚持“四重点一稳定一保证”工作总格局，坚持目标导向、问题导向和“创优势、增实力、补短板、能抓住”工作方针，落实好“党委加强领导、政府充分履职、人大政协共同参与”责任明晰、工作有序、运转高效的工作机制，坚持以项目为带动、项目化推进，明确任务书、时间表和路线图，确保定一件、干一件、成一件。

——站在国家中心城市高质量建设新的历史起点，我们要保持竞进姿态。要树立世界眼光，围绕“整体工作创一流、重点工作做示范、特色工作争先锋”政府工作目标，身体力行“四问、四标”工作要求： “四问”： 即围绕国家中心城市建设，一问思想站位高不高，是不是世界眼光？二问研究谋划深不深，是不是国际标准？三问方法路径对不对，是不是改革创新？四问措施作风实不实，是不是高效务实？着力以“四问”推动经济高质量发展、城市高品位建设，形成交通枢纽、开放平台、经济体系、营商环境、城市风貌的“五个国际化”。 “四标”： 即寻标、对标、达标、夺标，找差距、补短板、创优势，每项工作都要对标国内外先进城市找标杆、对标杆、追标杆、超标杆，力争在国家中心城市建设中先出彩、出重彩。

今年政府工作的总体要求是：坚持以习近平新时代中国特色社会主义思想为指导，全面贯彻党的十九大和十九届二中、三中全会及中央经济工作会议精神，深入贯彻习近平总书记视察指导河南和郑州时的重要讲话精神，认真落实省委十届八次全会、市委十一届九次全会部署，牢固树立新发展理念和以人民为中心的发展思想，把握“稳中求进”总基调、突出“奋发有为”总要求，以高质量发展为根本方向，以供给侧结构性改革为主线，以国家中心城市建设为统揽，以航空港实验区建设为引领，以“五区联动”“四路协同”为突破，坚持“四重点一稳定一保证”工作总格局，坚持目标导向、问题导向和“十二字”工作方针，坚持项目带动、项目化推进工作，继续打好三大攻坚战，统筹推进稳增长、促改革、调结构、惠民生、防风险各项工作，进一步稳就业、稳金融、稳外贸、稳外资、稳投资、稳预期，提振信心、激发活力，保持经济持续健康发展和社会大局稳定，推进国家中心城市建设迈出更大步伐，为全面建成小康社会收官打下决定性基础，以优异成绩迎接新中国成立70周年。

主要预期目标：生产总值增长8%左右；规模以上工业增加值增长7%左右；固定资产投资增长8%以上；一般公共预算收入增长8%左右；税收占一般公共预算收入比重达到75%左右；社会消费品零售总额增长9.5%；进出口保持平稳增长；居民人均可支配收入增长8%；居民消费价格指数涨幅控制在3%左右；常住人口城镇化率提高1.3个百分点以上，全社会研发投入强度达到2%；城镇调查失业率、登记失业率控制在5.5%、4%以内；节能减排、环境保护等指标完成国家、省下达任务。

三、2019年重点工作

2019年，我们将紧盯国家中心城市高质量建设总目标，紧紧围绕“让郑州这个龙头高高扬起来”新要求，加快“一中枢一门户三中心”建设，突出抓好十个方面工作。

（一）着力构建现代产业体系，加快建设国家新的经济增长中心。坚持稳增长、促转型、重民营、强县域、活金融相结合，夯实经济高质量发展的基础支撑。

深化供给侧结构性改革。坚持巩固、增强、提升、畅通“八字”方针，一是巩固“三去一降一补”成果，推动产能过剩行业加快出清，促进土地、资本、人才等要素向优质企业、新兴产业流动。二是增强微观主体活力，发挥企业和企业家主观能动性，构建以公平竞争、优胜劣汰为主导的正向激励机制，破除各类要素流动壁垒。三是提升产业链水平，加快构建以信息、技术、知识、人才等要素为支撑的竞争新优势，促进产业迈向全球价值链中高端水平。四是畅通经济循环，强化产销、产需、产运对接，提高金融体系服务实体经济能力，着力建设统一开放、竞争有序的现代市场体系。

牢牢扭住稳增长基本要求。一是突出强投资。坚持民间投资和政府投资“两手抓”，紧盯总投资2.13万亿元的910个省市重点项目，确保完成投资4600亿元以上；落实国家、省、市鼓励民间投资政策措施，为民间投资营造良好环境。实施工业“1312”强投资计划，推进上汽大数据中心等200个项目开工建设，加快推动宇通电动客车技术中心等200个以上项目竣工投产。二是着力稳实体。全面落实国家减税降费等政策，清理规范各类行政审批中介服务事项，全面清理涉企收费，加强运行监测预警分析，强化电力、运力、能源等要素保障，突出稳定工业增长。三是积极扩需求。紧跟国家政策导向促进消费升级，充分释放健康、文化、信息等领域和传统服务业消费潜力，落实好个人所得税专项附加扣除政策，积极培育新的消费热点。四是强力抓招商。落实招商引资“一把手”工程，深度对接世界500强和国内外行业领军企业，大力实施精准招商，定向引进一批建链、延链、强链、补链企业和项目，力争实际到位资金2450亿元，其中实际利用外资43亿美元，新增世界500强企业不少于5家。

全方位支持民营经济发展。像爱护眼睛一样爱护民营经济，像对待亲人一样善待民营企业。全面落实省“非公经济20条”，优化市“民营经济40条”，坚持“两个毫不动摇”，促进多种所有制经济共同发展。积极营造公平、透明、高效的普惠性市场环境，构建“亲清”新型政商关系，研究解决民营企业发展中遇到的困难，切实依法保护企业家人身和财产安全。市场经济是企业家经济，企业家是郑州最宝贵的战略资源，我们要为企业提供“六心”服务，充分激发郑商活力，让广大企业家坚定信心向前走、心无旁骛谋发展。

加快推进产业转型升级。以高端化、绿色化、智能化、融合化为方向，一是强化智能制造引领。实施电子信息、汽车、装备制造等5个重点产业转型升级行动计划，智能传感器、信息安全等5个新兴产业培育专案，加快建设信息安全产业基地和智能传感谷，着力培育世界级智能终端、客车、超硬材料等先进制造业集群；加快铝及铝精深加工、家居和品牌服装制造等传统产业改造升级。深入推进质量强市战略，推动经济转型发展；实施制造业研发投入三年倍增计划，加快制造业创新中心、产业创新联盟等创新平台建设。推进制造业和服务业融合，加快工业互联网平台建设，持续推进“百千企业上云”计划。实施“三大改造”，力争技改投资增长30%以上。积极筹办中国服务型制造大会和第二届世界传感器大会，利用好2019年两岸智能装备制造郑州论坛、豫沪产业合作对接活动等平台，力争签约亿元以上项目100个以上、签约额1500亿元以上。实施战略性企业培育、企业家领航、中小企业培育等行动计划，推动百家企业“三年倍增”发展，力争百亿级工业企业达到14家。积极推进5G技术应用、物联网应用、人工智能应用，抢占数字经济制高点，加快建设网络强市。二是推进现代服务业提质。加快区域金融中心建设，力争金融业增加值突破1200亿元。建设国家现代物流创新发展试点城市，力争物流业增加值达到850亿元。大力发展健康养老服务，力争新增养老床位4000张，加快推进郑州宜居健康城、河南精准医疗产业园等项目建设。积极吸引国际展会活动来郑举办，力争展览总面积增长6%以上，国际性展会达到12个。坚持“房子是用来住的，不是用来炒的”定位，加快建立租购并举住房制度，构建房地产平稳发展长效机制。大力支持技术、信息等专业服务业，着力发展供应链管理、信息增值等服务型制造新业态。三是促进都市现代农业增效。打造 “一带四区五组团”格局，加快建设环城都市生态农业圈和沿黄生态涵养带。实施生态农业建设等“四大工程”，力争新发展环城都市生态农业10万亩、湿地农业和高标准“菜篮子”生产示范基地各1万亩、培育全国休闲农业与乡村旅游星级企业10家。同时，继续抓好“大棚房”清理整治。持续抓好非洲猪瘟疫情防控。筹办好第20届中国绿色食品博览会。

用好资本市场的金融活水。一是发挥国家中心城市建设产业发展基金和投融资担保专项资金作用，支持制造业高质量发展。用足用活地方政府债券政策，支持发行企业债券、双创专项金融债券和孵化专项债券，激发民间投资活力。二是优化金融服务，大力发展绿色金融、普惠金融、科技金融，常态化办好重大项目融资对接会，推动郑州银行、中原银行、郑州农商行等提高存贷比，打通企业融资“最后一公里”，降低民营企业获得信贷难度和成本。三是实施企业挂牌上市和并购重组提速计划，持续打造上市挂牌“郑州军团”、并购重组“郑州高地”、资本市场“郑州板块”，力争上市公司和新三板挂牌公司达到220家。

强力推进县域经济高质量发展。全面贯彻习近平总书记县域治理“三起来”、乡镇工作“三结合”重要指示，加快组团式发展，明晰发展定位，放大比较优势，彰显县域发展特色。扎实开展产业集聚区、服务业“两区”高质量发展专项行动，支持各开发区、县（市）区培育1-3个主导产业，打造一批具有国际国内竞争力的千亿级产业集群，做大做优做强县域经济“单元格”。

（二）着力实施创新驱动发展战略，加快建设国家创新创业中心。突出企业创新主体地位，加快培育创新生态链，汇集改革动力、激发人才活力，真正让创新成为引领发展的第一动力。

持续提升科技创新能力。一是全力支持自创区发展，谋划建设自创区展示中心，积极打造中原科创谷、郑东新区科学谷、郑开双创走廊。二是加大政策支持力度，加强专利权、商标权、版权等要素市场和产权市场法治建设。研究建立助推高质量发展的科技创新政策体系，大力培育高新技术企业，积极引进培育雏鹰企业、瞪羚企业、独角兽企业。三是深入推进“政产学研用”协同创新，充分发挥国家技术转移郑州中心、河南省技术产权交易所等作用，加速科技成果转化。加快推进国家超算中心、中科院过程所郑州分所等项目建设。组织实施盾构、燃料电池客车等50个科技创新重大专项。办好中国·郑州北斗应用大会、独角兽企业峰会、新兴产业大会、中国·河南开放创新暨跨国技术转移大会、双12双创日暨第五届中国创客领袖大会等活动。

扎实推进军民融合深度发展。全力推进6个国防科技关键核心“卡脖子”技术攻关，加快开发和实施一批军民两用技术产品。依托新材料、精密机械加工、高端装备制造等军民融合重点领域，有效引导“民参军” “军转民”。加强与省内外央属军工单位对接，加快引进综合实力强、科技含量高的军民融合型企业。加快建设河南郑州军民融合产业创新基地（荥阳）、河南军民融合产业基地（巩义）、北斗产业园（高新区）。

加大“四个一批”培育力度。积极引进培育一批行业领域隐形冠军企业，力争新培育高新技术企业500家；持续推进大中型企业研发机构全覆盖工程，加速布局市级创新平台；引进建立新型研发机构10家以上，高标准建设中科院计算所郑州分所、浙江大学中原研究院、同济大学中原环保产业研究院、固高郑州智慧产业研究院；积极打造国际人才新高地，大力实施“智汇郑州”人才工程，出台更具竞争力的人才政策，加快推进设立海外人才工作站，着力引进一批站在世界科技前沿、处在创新高峰期的领军人才和创新团队，力争引进领军人才和高层次紧缺人才200名。

强力打造“双创”升级版。健全完善人才培养、评价和激励机制，探索实施顶尖人才“全权负责制”，落实科研人员发明成果转化创业、科技成果收益分配、股权期权激励等政策，推动科研机构与企业双向交流，培养一批为企业提供智力支持的科研人才团队。高质量建设各级各类双创示范基地、大学生创新创业实践示范基地、小型微型企业创业创新示范基地、创业孵化园等，支持社会力量多元建设创新创业综合体；着力打造“郑创汇”“强网杯”等创新创业大赛品牌。力争万人发明专利拥有量达到16件，科技进步贡献率达到64%。

（三）着力提升城市文化软实力，加快建设华夏历史文明传承创新中心。围绕“建设国际文化大都市，打造中华文明与世界文明交流对话重要平台”，打造城市名片，提升文化品位，塑造城市形象，不断提升文化对全球吸引力、影响力和传播力。

高标准建设重点片区。一是抓好中央文化区（CCD）。加快CCD区域重大公共服务平台建设，打造一批具有郑州特色的文化新地标。确保奥体中心、大剧院、新博物馆等项目建成投用，全面加快北部片区建设步伐。二是抓好四大历史文化片区。开工建设商都历史文化区公建类项目和商业文化片区，全力打造中原地区“城市会客厅”。大力提升百年德化历史文化片区德化街环境，着力打造城市文化“品牌工程”。加快建设二砂文化创意园，着力打造国内一流“城市创新工场和工业历史博物馆”。加快推进荥泽古城一期文化片区、水系等重点项目建设，努力打造中华文明史诗级地标、国际知名旅游目的地。四大片区完成投资100亿元以上。

推动文化事业繁荣发展。一是实施文化惠民、文化精品工程。积极引导城乡居民扩大文化消费，建设“绿城智慧书房”50处；推进县乡文化馆图书馆总分馆制，新增分馆15个；组织好“千场演出”“万场电影”“绿色周末”、精品剧目演出季等文化惠民项目；打造提升《精忠报国》《朝阳城》《郑风·意象》等新剧目，努力争取国家艺术奖项。围绕庆祝新中国成立70周年等重大节日，组织开展各类丰富多彩的群众文化活动。二是加强历史文化遗产保护利用。编制全域文物保护利用示范区总体规划，打造国家文物保护利用示范区。新建生态保遗项目22个。郑州博物馆新馆和“两院”建成开放，三年内谋划建设各类博物馆100家以上。实施中原文物全媒体传播计划。启动文物安全智慧监管平台建设。加强非物质文化遗产名录体系建设，鼓励扶持民营展示馆、传承基地建设。

培育文旅产业集群优势。一是加快“只有河南”主题演艺公园、宋城·黄帝千古情、银基佳宝乐园、列子小镇等一批重点项目建设，推进文化传承保护创新，培育壮大文化消费市场。二是广泛吸收世界先进文化元素，赋予新时代内涵和现代表达形式，精心筹办己亥年黄帝故里拜祖大典，加快建设中牟国际文化创意园区，依托华强中华复兴之路、电影小镇、海昌海洋公园等项目，全面打造“东方奥兰多”。 三是促进旅游业转型升级，做大做强“功夫郑州”、黄帝文化、黄河文化、嵩山文化、河洛文化等知名品牌，加快构建快进慢游新格局，力争跻身国内旅游城市第一方阵。

（四）着力打造内捷外联“畅通郑州”，加快建设国际综合交通物流中枢。突出抓好九大重点工程，巩固扩大提升“域外枢纽、域内畅通、多式联运”三大优势。

一是抓好航空枢纽能级提升工程，以郑州国际航空货运枢纽战略规划获批为契机，加快推进机场三期工程建设，完善中央航站区布局，提升机务维修等配套能力，打造24小时内全球可达的航空服务体系。二是抓好米字形高铁网实施，配合做好郑万、郑阜、郑济高铁和郑州南站建设，确保郑万一期、郑阜高铁年底前通车运营。三是抓好铁路货运体系建设工程，持续推进占杨、薛店和上街铁路物流基地以及郑州南站高铁快运基地等工程建设，加快构建“1+2+N”铁路货运体系。四是抓好“1+4”郑州大都市区交通构建工程，完善郑州与周边地市“3+3+4”快速交通系统，确保机场至南站城际铁路建成通车，开工建设机登洛城际铁路，争取开工建设焦平高速（郑州段），加快连霍高速外移等工程前期工作。五是抓好环城货运通道建设，推动107国道东移与四港联动大道连接线等6个项目完工。六是抓好“米字+环线”轨道交通建设工程，力争4月底前轨道交通第三期建设规划获批，5号线5月份通车运营，市民文化服务区地下交通市政工程、2号线二期、城郊线二期等线路年底前投用；3号线一期、4号线等加快建设。七是抓好城市路网建设工程，金水路西延、农业快速路铁路代建部分、四环线及大河路快速化高架主线等工程投用，开工建设紫荆山路-长江路综合立交等一批快速路工程，续建新建支线道路55条，基本消除三环内断头路。八是抓好公交场站和公交专用道建设工程，积极发展“全域公交”，巩固提升“公交都市”创建成果。九是抓好多式联运“一单制”工程，围绕物流枢纽承载城市定位，提升公铁集疏、陆空衔接、铁海联运水平，研究建立“一单制”联运体系，构建一站托运、一次收费、一单到底的现代国际物流中心。

（五）着力推动“五区联动”“四路协同”，加快建设内陆地区对外开放门户。加强政策研究、制度创新，积极融入全球经济体系，巩固提升“一带一路”核心节点城市地位。

全面提升“五区联动”优势。一是强化航空港实验区引领作用。开工机场三期北货运区，加快智能终端及新型显示、智能装备、生物医药、航空制造和服务、航空物流等产业发展。二是高水平建设自贸区郑州片区。围绕“两体系一枢纽”定位，深入推进政务、金融、监管、法律、多式联运五大专项体系建设，力争256项改革创新任务和153项复制推广任务基本完成，持续推进申建自由贸易港。三是全力支持自创区发展。深化管理体制和人事薪酬制度改革，启动自创区核心区扩区工作。四是加快推进跨境电商综试区建设。加快EWTO核心功能集聚区专项政策的制定落实，打造跨境电商零费区；积极申建跨境电商进口药品监管服务试点；反向复制1210进口模式，开行直飞欧美包机，举办第三届全球跨境电商大会。五是加快国家大数据综试区建设。以龙子湖智慧岛为中心，辐射高新区、金水科教园区，力争入驻大数据企业300家以上、产值超过300亿元。

巩固扩大“四路协同”效应。一是推动“空中丝绸之路”强基扩面。加快组建本土客货运航空公司，推动国内外知名航空物流服务企业落地。深入实施郑州-卢森堡“空中丝绸之路”专项规划，推动第五航权业务开展，加快组建卢森堡合资货航公司。支持卢森堡货航开辟1-2条洲际货运航线，增加1-2个通航点。力争客运量突破3000万人次，货邮吞吐量达到52万吨以上。二是推动“陆上丝绸之路”扩线提量。开通经满洲里、绥芬河口岸线路，力争中欧班列（郑州）全年开行1300班以上。三是推动“网上丝绸之路”便捷便利。完善跨境电商供应链体系，拓展跨境电商出口业务，引进跨境电商大型平台类、物流类、支付类总部企业，力争跨境电商交易额增长20%。四是推进“海上丝绸之路”无缝衔接。打造“郑州港”内陆港，通过海铁联运、公海联运等方式让港口功能平移至“郑州港”，力争在郑州海关报关放行不少于1万个标准集装箱。

切实加强开放平台建设。一是抓好口岸和海关特殊监管区建设，加快集装箱中心站第二线束、汽车口岸二期项目建设，加快推进汽车平行进口试点，建设“一站式”大通关服务体系，打造智慧口岸，积极推进建设海外仓及物流分拨中心。二是加快实施《郑州市推进中欧区域政策合作案例地区建设工作方案（2018-2020年）》，持续提升城市国际化水平。三是着力推动外贸转型升级，促进跨境电商与制造业融合发展，尽快把产业优势转化为出口优势，力争全市外贸进出口总额完成4100亿元以上。

（六）着力破解全面建成小康社会瓶颈制约，坚决打好打赢三大攻坚战。切实防范化解重点领域风险。坚持消存量、控增量并举，加快非法集资案件办理，构建互联网金融监管长效机制。探索建立部门联动、上下联手、市场运作机制，持续推动上市公司流动性危机化解。强化债务风险管控，严守政策红线，依法规范政府举债行为，有序稳妥化解债务风险。建立专班专案，有效化解房地产领域问题楼盘。强化应急管理，提升防灾减灾救灾能力，压实安全生产责任，全面推行双重预防体系建设，坚决防范遏制各类事故发生。做好突发公共事件预防处置，有效防范社会风险。

坚决打赢脱贫攻坚战。落实脱贫攻坚三年行动实施方案，解决好“两不愁三保障”突出问题，切实增强贫困群众内生动力和自我发展能力。把防止返贫摆在更加突出位置，狠抓政策举措落地、就业产业带动、综合保障兜底，持续巩固扩大脱贫成效。扎实推进易地扶贫搬迁后续扶持和黄河滩区居民迁建，确保搬迁群众“稳得住、能致富”。持续做好结对帮扶贫困县工作。

全力打好污染防治攻坚战。强化“党政同责、一岗双责”“三管三必须”，强力推动生态环境保护再上新台阶。一是坚决打赢蓝天保卫战。持续优化能源结构，加快推进主城区煤电机组清零工程，推动煤炭消费总量稳步下降；加快调整产业结构，实施重污染企业退城搬迁、重点企业“一企一策”深度治理改造；积极调整交通运输结构，继续实施公铁联运，加快建设城市物流配送体系，建设完善“天地车人”一体化的机动车排放监控系统，打好柴油货车污染治理攻坚战；不断提高扬尘污染治理标准，持续抓好“六控”，确保全市PM10、PM2.5年均浓度、城区优良天数达到省定目标。二是坚决打好碧水保卫战。加强全市河湖管理保护，深化落实河（湖）长制，持续推进“三污一净”专项治理；深入开展河流综合整治、黑臭水体整治、打击非法采砂、全域清洁河流和农业农村污染治理攻坚战；实施重点行业提标治理，加快污染处理设施建设提标；持续开展饮用水源地保护攻坚战，确保水源地水质稳定。三是强力推进净土保卫战。强化土壤污染源头防治和重点企业监管，加强重金属污染防治，建立更新污染地块清单和优先管控名录，实施污染地块动态化管理，有序开展污染土壤修复与治理试点工作。

（七）着力塑造现代化城市形态风貌，扎实推进城乡一体化建设。树立精明增长的城市发展理念，提升城市功能品质，大力实施乡村振兴战略，持续推进以人为核心的新型城镇化。

提升城市规划建设管理水平。一是发挥规划引领作用，推动“多规合一”，高质量、高标准完成郑州市城市总体规划（2018-2035年）及综合交通体系规划、轨道交通线网规划修编等15项专项规划。建立国土空间规划体系，优化国土空间开发保护格局。二是大力实施精准供地，推进批而未供和闲置土地专项整治，强化自然资源要素保障，落实最严格的耕地保护制度。三是以文明城市创建为抓手，编制出台《城市精细化管理白皮书》，持续推进三级“路长制”，引入第三方评估机构，以绣花功夫、匠心精神促进城市管理规范化、标准化、精准化、智慧化。四是全面启动楼体广告、门头牌匾整治，持续推进灯光亮化工程，对62条道路实施中修和人行道提升改造，推进架空通信线缆入地改造、交通秩序、市容环境和城乡结合部综合整治，彻底改善城乡结合部48个乡（镇、办）脏乱差面貌。五是推进垃圾分类处置，生活垃圾分类覆盖率达到70%以上、回收利用率30%以上，建筑垃圾资源化利用率65%以上。六是加快标准化市场建设和提升改造力度，持续推进市场外迁，推动商品交易市场转型升级。

深入推进百城建设提质工程。一是市县协同推进，支持六县（市）围绕打造全国一流县级城市目标，与文明城市创建等融合推进。二是坚持“补短板、强功能、增效能、提品质”，全面落实城市“四篇文章”和城市“四治”专项行动方案。三是切实加强楼院治理，加快推进以老旧小区整治提升为重点的老城区有机更新，实现无主管老旧小区物业全覆盖，力争2年内完成整治提升任务。四是加快提升23个中心镇综合承载能力和服务功能，发挥好在县域城镇体系中的节点支撑作用。加快构建网络化、无障碍、功能复合的公共服务网络，推进15分钟城乡社区生活圈建设。五是加快推进“城市双修”、综合管廊、城市设计、海绵城市等试点城市建设；政府投资新建公共建筑实行超低能耗建筑标准，民用建筑大力实施绿色建筑标准。六是积极推进既有住宅加装电梯和公厕建设改造，新建公厕393座、达到每平方公里6座。

切实加强基础设施建设。加快推进次高压燃气管道及配套调压站、豫中LNG应急储备中心等项目建设，新建改建燃气管网100公里，发展天然气用户10万户；抓好清洁取暖示范城市试点建设，开工建设新密裕中电厂2台100万千瓦供热机组“引热入郑”集中供热配套管网工程，新建改造供热管网63公里，新增集中供热面积500万平方米。确保东部垃圾焚烧发电厂建成投用，推进西部垃圾焚烧发电项目、华润登封电厂西热东送“引热入郑”等项目尽早开工。加快建设郑州新区污水处理厂二期等项目，新增污水处理能力15万吨/日、污泥处理能力300吨/日，中水利用率提高到45%。积极推进500千伏建新变（电站）等70个项目开工，500千伏惠济变（电站）等35个项目投运，完成295个行政村电网改造。

加快建设新型智慧城市。坚持“善政、惠民、兴业”方向，抓好智慧交通、城管、教育、医疗等规划编制实施，完善郑州政务云、城市大脑一期等基础设施，加快推进全市统一的党政政务办公系统、“无现金支付结算城市”“i郑州APP”、智慧停车等重点示范应用项目，持续推进郑州直联点提升工程，加快中国联通中原数据基地二期、中国移动（河南）数据中心一期等重点项目建设。

深入实施乡村振兴战略。统筹推进“五个振兴”，努力在乡村振兴中走前列、做示范。一是以农民增收为导向，推进乡村产业跨界融合，启动“农村集体经济+农商文旅体融合发展”试点。二是以改善环境为突破，突出抓好农村生活垃圾和污水处理、“厕所革命”等工作，具备条件村庄实现生活污水集中处理设施全覆盖，建设美丽乡村试点村32个，启动城乡融合共享特色田园乡村建设项目6个。三是以土地制度改革为牵引，深化农村集体资产股份化改革，健全农村产权交易服务机制，稳步推进农村宅基地和集体产权制度改革。四是以基层党建为重点，实施村党组织带头人优化提升行动，突出政治功能，强化服务功能。鼓励各类人才返乡创业，培育一批新型职业农民和乡村急需紧缺高端人才。

（八）着力全面深化改革，激发经济社会发展内生动力。突出抓好已出台改革举措落实，进一步优化营商环境、提升行政效能、激发市场活力。

加快构建国际化法治化便利化营商环境。把优化营商环境作为经济高质量发展、国家中心城市高质量建设的生命线，统筹推进政务、商务、市场、人文、法治、城市“六大环境”建设，扎实开展开办企业、贸易便利化等10个对标提升专项行动，实施营商便利度评价指标体系，清除一切影响投资自由化、贸易便利化的障碍，力争营商环境全省第一、进入全国重点城市前列，各开发区、县（市）区全部处于省第一方阵，自贸区郑州片区成为全国营商环境“排头兵”。新增市场主体20万户以上。

持续深化“放管服”改革。开展“最多跑一次”改革规范提升年活动，围绕“马上办、就近办、网上办、一次办”，做好办事大厅管理水平提升、基层政务服务全覆盖、重点领域改革攻坚等工作，打通政务服务的堵点难点，努力让群众办事不作难。力争市县乡办事大厅审批服务事项“应进必进”，100个高频事项“最多跑一次”，50个事项凭身份证直接办理，群众办事申请材料削减60%以上，企业投资项目审批控制在100个工作日以内。

深入推进重要领域和关键环节改革。完成政府机构改革，优化职能配置，提高行政效能。深化投融资体制创新，用好政府债券，完善政府和社会资本合作政策体系。继续抓好国资国企改革，稳妥处置“僵尸企业” ，加大剥离企业办社会职能力度，深化企业产权结构、组织结构、治理结构改革，全面加快市直部门监管的市属企业改革。积极发展混合所有制经济，建立完善差异化薪酬分配和长效激励约束机制，加快实现从管企业向管资本转变。深化财政事权与支出责任划分、全过程预算绩效管理改革。大力推进要素市场化配置改革，积极推进执法体制、信用管理体制、价格、科技等领域改革。

（九）着力强化生态文明建设，打造生态宜居“美丽郑州”。深入实施生态文明建设“四大行动”，全力打造天蓝地绿水净、宜居宜业宜游的美丽中国示范城市。

加快建设森林生态系统。大力实施国土绿化提速行动，围绕“六化”目标，完成营造林16万亩，加快郑州树木园改造提升、侯寨森林公园、邙岭森林公园等重大项目建设，大力发展特色经济林、花卉苗木、森林旅游等特色林业产业，着力打造“森林郑州”。

加快建设城市生态系统。大力推进国家生态园林城市创建工作，建设公园城市，实施“300米见绿、500米见园”三年建设规划；不断完善城市公园体系，中心城区公园围墙应拆尽拆，今后中心城区拆迁区域面积10亩以下的地块，全部建成公园绿地；在城市外围开工建设郊野公园10个，建成3-5个；做好郑州第二动物园、第二植物园前期准备工作；制定实施城区绿化导则，大力开展社区周边、重要节点微景观打造；持续推进铁路沿线、国省干道、高速出入口等交通路网绿化提升工程，高标准打造绿色视觉通廊。

加快建设湿地生态系统。加强黄河湿地保护修复，推进黄河滩区生态环境综合治理，规划建设郑州黄河中央湿地公园。积极开展省级湿地公园建设，推进荥阳万亩湿地等一批人工湿地公园建设。加快推进湿地农业建设。

加快建设农田生态系统。扎实推进农林复合生态系统规划与建设，积极实施畜禽生态养殖、土壤面源污染、农村人居环境三项综合治理，大力改善农业生态环境，不断优化农业生产条件，大力调整种植结构，提升农田生态系统综合服务功能。

加快建设流域生态系统。坚持“四水同治”。实施国家节水行动。强力推进水生态重点项目建设，贾鲁河（包括西流湖）综合治理蓝线工程基本建成，绿线工程主体完工，西流湖段按照时间节点完成任务，牛口峪引黄工程、环城生态水系循环工程等建成通水；加快推进古汴河疏浚和金水河、熊儿河、溱水河整治提升工程，启动实施贾峪河常庄水库坝后段生态治理工程（水博园一期）、贾鲁河尖岗水库大坝至南四环桥段治理工程，谋划推进西水东引等工程前期工作。

（十）着力保障和改善民生，切实让群众共享改革发展成果

继续办好民生实事。一是全市新增城镇就业11万人，农村劳动力转移就业4万人。建成保障性住房5万套，分配公租房7500套，回迁安置群众15万人，完成安置房网签9万套，建成投用便民服务中心50个。二是全市新增公办幼儿园100所，市区新建、改扩建中小学校30所、新投用中小学校20所。三是免费为具有郑州市户籍的适龄妇女进行“两癌”筛查、为孕妇进行唐氏筛查和产前超声筛查、为新生儿进行听力障碍初筛等疾病隐患筛查等。四是开展“舞台艺术进乡村、进社区”文艺演出1200场；引进、组织精品剧目（节目）25台；建成基层综合性文化服务中心100个。五是新建、提升改造标准化农贸市场20家；新创建食品安全管理示范单位、餐饮示范店各100家；在全市幼儿园建成明厨亮灶1200家。六是购置新能源公交车1000台，建成充电站28座；市区新增公共停车泊位5万个以上；建成新能源汽车充电桩5000个；新改建“四好”农村公路130公里；完成农村户用卫生厕所改造10万户。七是对符合条件的视力、听力、言语残疾人，每人每月发放30元通讯信息消费补贴；新增50个、提升100个“绿城妈妈”社区环保服务项目；新增、提升“儿童之家”各50个；将儿童免费乘坐公交的身高标准由1.2米提高到1.3米。八是新建、改建城镇社区多功能运动场、社区健身活动中心、农民体育健身升级工程300个，新增健身路径300条。九是建成公园、游园、微公园400个，连通提升生态廊道400公里，市区新增绿地面积1000万平方米。

坚持稳定和扩大就业。把稳就业放在更加突出位置，积极推进公共服务专项行动，突出做好高校毕业生、城镇困难人员、退役军人等重点群体就业工作。持续实施全民技能振兴工程，开展各类职业培训33万人次。完善政府贴息创业担保贷款机制，支持各类群体创业。切实做好过剩产能化解中职工安置工作。支持农民工等群体返乡下乡创业，实施失业保险援企稳岗“护航行动”。

稳步提高居民收入水平。贯彻落实扩大中等收入群体规模政策措施和最低工资保障制度，加强对企业工资集体协商的分类指导，推动企业建立健全工资共决及正常增长机制。完善国有企业工资决定机制改革相关配套政策，建立健全企业薪酬调查和信息发布制度。跟进实施公务员地区附加津贴制度。

织密社会保障网络。一是继续提高退休人员基本养老金和城乡居民养老保险待遇水平。二是完善社会保险转移接续政策，开展新经济新业态灵活就业人员参加工伤保险国家试点。三是推进城乡低保标准一体化。做好城乡低保专项治理工作，健全完善临时救助制度。

持续提升教育发展水平。推进城镇小区配建幼儿园规范建设，完成100所义务教育学校标准化建设，市区新开工建设外国语学校港区校区、回中港区校区、二中经开区校区等4所普通高中，努力消除城镇义务教育阶段大班额等突出问题。强力推进职业教育优质发展，开展国家产教融合试点城市申报工作；加大对市属高校支持力度，持续支持郑州大学“双一流”建设。加快推进国内外优质高等教育资源来郑办学。加强教师队伍建设，改善教师工作生活条件。

加快建设健康郑州。完善公共卫生服务体系，加大传染病、慢性病、地方病、职业病等防治管力度。健全健康服务管理网络，推进城企联动、普惠养老，努力提高群众健康保障水平。全面深化59家公立医院综合改革，加快推进国家区域医疗中心建设，扎实推进15家县级医院基础能力和急诊、心血管等34个县级临床重点专科建设。加强分级诊疗制度建设，推进县域“医共体”、社区卫生服务中心建设。推进按病种收付费改革，完善药品供应保障制度。实施中医药振兴发展工程。积极创建全国全民运动健身模范城市，加快构建城市15分钟健身圈，培育郑州体育活动品牌，引入和培育6个以上国际性、国字号文化体育活动项目落户郑州，着力打造体育强市。继续办好郑州国际马拉松等大型赛事；集全市之力高质量办好第十一届全国少数民族传统体育运动会。

深化社会治理创新。借鉴“街乡吹哨、部门报到”改革经验，以党建引领基层治理创新。强化正面宣传及政策解读，针对社会重大关切点，及时完善政策举措，合理引导预期。深化市场监管体制改革，加快构建现代化市场监管体系，建立食品药品智慧监管平台，积极创建食品安全示范城市。健全预防和化解社会矛盾机制，高质量推进“一村（格）一警”工作，加强信访法治化建设，夯实平安郑州建设基础。切实做好农民工工资治欠保支工作，积极维护农民工合法权益。深入开展“扫黄打非”。提高网络治理能力，着力打造清朗网络空间。推动“扫黑除恶”专项斗争纵深发展，切实做好重要时间节点安全稳定工作。

积极践行社会主义核心价值观，持续开展精神文明创建活动。加强国防动员、后备力量和人民防空建设，深入开展“双拥”共建。加强民族宗教工作基层基础建设，促进民族团结、宗教和睦。加强统计工作，扎实开展第四次全国经济普查。做好外事、侨务、港澳、对台工作，抓好审计、机关事务、地方史志、档案、气象、地震、移民安置、援疆等工作。积极支持工会、共青团、妇联、科协、文联、侨联、社科联等群团组织工作。

四、全面加强政府自身建设

民之所望，政之所向。我们要始终坚持以人民为中心的发展思想，砥砺前行不懈怠、争先进位谋出彩，以想事、谋事、干事、成事的过硬作风，展示新形象、开创新局面。

（一）坚定信念务实干。坚持以习近平新时代中国特色社会主义思想武装头脑、指导实践、推动工作，树牢“四个意识”、坚定“四个自信”，做到“两个维护”。始终对习近平总书记步伐步步紧跟，对党中央决策部署闻令而动，把党的要求贯彻到政府工作全过程、各领域，确保政令畅通、令行禁止。始终涵养“功成不必在我”精神境界，葆有“功成必定有我”历史担当，发扬钉钉子精神，一张蓝图绘到底、一锤接着一锤敲，苦干实干、久久为功。

（二）依法行政规范干。全面贯彻依法治国方略，持续推进“七五”普法，切实加强法治政府和诚信政府建设。坚持尊法学法守法用法，善于运用法治思维和法治方式深化改革、推动发展、化解矛盾、维护稳定。完善政府法律顾问制度，健全政府咨询专家库，推进决策法制化、科学化、民主化。深入推进行政机关负责人出庭应诉，认真执行法院生效裁判。严格执行市人大及其常委会决议决定，自觉接受人大法律监督、工作监督和政协民主监督，自觉接受监察机关监督，加强审计监督，主动接受社会监督和舆论监督。完善政府立法机制，严格规范公正文明执法，防止任性用权，决不允许以权代法、以权压法、逐利违法、徇私枉法。

（三）牢记宗旨清廉干。始终与人民群众站在一起、想在一起、干在一起，把人民群众的难点痛点作为履职重点，为人民群众全心全意服务、真心真意办事。严格遵守廉洁自律准则，始终敬畏人民、敬畏组织、敬畏法纪，坚决贯彻中央八项规定及实施细则精神，聚焦突出问题、紧盯关键节点，下大力气纠“四风”、树新风，坚定不移反腐倡廉，深入抓好以案促改，让钢规铁纪成为政府工作人员的自觉遵循。

（四）主动担当创新干。始终保持融入新时代、奋斗新时代的姿态，冲破思想观念束缚，突破利益固化藩篱，改革开放再出发，对发展负责、对历史负责、对人民负责，不避矛盾，不惧挑战，不推责任，勇挑重担。全面锤炼“五个过硬”、增强“八种本领”，强化正向激励，坚定使命必达的决心和舍我其谁的担当。坚持厚爱严管结合、激励约束并重，理解基层、关心基层，坚决把容错纠错机制落到实处，为担当者担当、为负责者负责、为实干者撑腰，让担责担难担险成为郑州干部最鲜明的底色。

各位代表！奋进新时代，追梦新征程，惟拼搏者强，惟奋斗者赢。让我们高举习近平新时代中国特色社会主义思想伟大旗帜，在省委、省政府和市委的坚强领导下，以锐意进取、永不懈怠的精神状态和敢闯敢干、一往无前的奋斗姿态，奋力谱写新时代郑州国家中心城市高质量建设新篇章，以优异成绩向新中国成立70周年献礼！
